# Machine learning for the prediction of three-year survival in locally advanced breast cancer patients receiving neoadjuvant chemotherapy using quantitative ultrasound imaging

**DOI:** 10.1038/s41598-026-51550-7

**Published:** 2026-05-05

**Authors:** Omar Falou, Lakshmanan Sannachi, Gregory J. Czarnota, Michael C. Kolios

**Affiliations:** 1 Department of Physics, Toronto Metropolitan University, Toronto, ON Canada; 2 Institute for Biomedical Engineering, Science and Technology (iBEST), Keenan Research Centre for Biomedical Science, St. Michael’s Hospital, Toronto, ON Canada; 3 Department of Radiation Oncology, Sunnybrook Health Sciences Centre, Toronto, ON Canada; 4 Department of Radiation Oncology, University of Toronto, Toronto, ON Canada; 5 Physical Sciences, Sunnybrook Research Institute, Toronto, ON Canada; 6 Department of Medical Biophysics, University of Toronto, Toronto, ON Canada

**Keywords:** Biomarkers, Cancer, Computational biology and bioinformatics, Oncology

## Abstract

Locally Advanced Breast Cancer (LABC) is a serious type of cancer with a poor prognosis despite advances in cancer treatment. As the disease is often inoperable, current guidelines recommend upfront aggressive neoadjuvant chemotherapy (NAC). While conventional ultrasound provides tissue echogenicity data, comparing images remains challenging due to the varied hardware configurations and instrument settings. Quantitative ultrasound (QUS) corrects this by using normalized power spectra calculations to derive quantitative parameters that are independent of instrument settings. In this work, we present an integrated deep-learning pipeline that reduces the possibility of data leakage and facilitates efficient data processing by combining scaling, oversampling, feature selection, and classification into a single framework. The pipeline is used to predict the three-year survival in LABC patients receiving NAC using QUS imaging before treatment initiation. The pipeline was trained on five quantitative ultrasound maps at the pre-treatment stage. The average acoustic concentration was the most predictive feature, achieving a recall and precision of 95% and 91%, respectively, for the survivor class. This work demonstrates that QUS may be used as a non-invasive biomarker for differentiating between LABC survivors and non-survivors at the pre-treatment stage. Prediction of the three-year survival rates of LABC patients before treatment can be used for prognosis, treatment planning, and patient decision-making.

## Introduction

Locally Advanced Breast Cancer (LABC) is a serious type of cancer with a poor prognosis despite advances in cancer treatment. LABC is characterized by tumors exceeding 5 cm that have spread to nearby lymph nodes but do not show distant metastasis^1^. According to the National Cancer Institute’s Surveillance, Epidemiology, and End Results (SEER), it is estimated that 20–30% of breast cancer cases may present as locally advanced, with an estimated 316,950 new cases of breast cancer expected in the U.S. in 2025^2,3^. As the disease is often inoperable, current guidelines recommend upfront aggressive neoadjuvant chemotherapy^4–7^. LABC patients have a 2- to 5-year survival rate of 30–60%, with many experiencing not only local recurrences but also metastatic progression^5^. These findings suggest that 40–70% of chemotherapy regimens are ultimately unsuccessful, indicating a lack of efficacy in both response rates and long-term survival outcomes. The three-year survival benchmark was selected for this study because it is clinically well-suited for evaluating LABC outcomes. At the three-year mark, both early recurrences and late treatment failures have typically manifested, providing a more comprehensive assessment of treatment efficacy than shorter endpoints (e.g., one or two years), which may overestimate treatment success by missing delayed progressions. Moreover, pathological complete response (pCR), a key prognostic indicator following NAC, has been most strongly correlated with overall survival at the three-year timepoint in published studies^8^. Conversely, extending the endpoint to five years introduces greater confounding from non-cancer causes of mortality, making the three-year mark an effective balance between clinical relevance and prognostic accuracy. Knowledge of the three-year survival rates of LABC patients who undergo neoadjuvant chemotherapy serves multiple purposes related to patient management, as well as treatment evaluation and public health. Survival rates provide key information to evaluate the effectiveness of current treatment strategies. Neoadjuvant chemotherapy treatment, which serves to reduce tumor size before surgical intervention, plays a vital role in determining long-term survival outcomes and assessing treatment protocols^9^. Research indicates that neoadjuvant chemotherapy leads to better overall survival rates, especially among patients who achieve a pathologic complete response (pCR)^8^. Survival statistics assist clinicians in making informed patient care decisions by providing prognostic information and allowing for treatment customization^10^. The survival data also help identify which demographic or clinical subgroups within the LABC population face an increased risk of negative results. Recent studies have shown that differences in age, alongside tumor subtype and socioeconomic status, influence survival outcomes^11,12^.

Imaging modalities such as MRI and PET have been employed as response prediction tools. They do, however, come with a high cost, long scan times, and the need for contrast agents^13^. A fast, inexpensive modality that does not require contrast agents would be highly valuable for predicting the response to neoadjuvant treatment. While conventional ultrasound provides tissue echogenicity data, comparing images remains challenging due to the varied hardware configurations and instrument settings. Quantitative ultrasound utilizes normalized power spectra calculations to derive quantitative metrics, including Average Scatterer Diameter (ASD), Average Acoustic Concentration (AAC), Midband Fit (MBF), Spectral Slope (SS), and Spectral Intercept (SI), for tumour characterization and treatment evaluation^14–17^. These metrics have been shown to correlate with patients’ responses to neoadjuvant chemotherapy (NAC), both before and after treatment initiation^18–21^.

Deep learning represents a branch of artificial intelligence which utilizes algorithms based on neural networks to process image data. Artificial neural networks operate as algorithms that bypass manual feature extraction by automatically learning essential patterns from raw data. The application of deep learning spans multiple image analysis tasks, including object detection, image segmentation, and classification. Recently, our group has demonstrated the potential of using transfer learning in predicting LABC response to neoadjuvant chemotherapy before the start of therapy using QUS imaging^17^. Our study analyzed QUS parametric maps from 174 patients acquired prior to treatment initiation by utilizing ResNet^22^ for feature extraction followed by feature selection and data balancing before classification. The model could differentiate between responders and nonresponders with a balanced accuracy of 88%. This work differs from our previous study in that the current pipeline integrates all preprocessing and classification steps (scaling, oversampling, feature selection, and model training) into a unified framework, thereby minimizing manual intervention and reducing the risk of data leakage through strict cross-validation practices. Specifically, by unified framework, we refer to the integration of all preprocessing and classification steps: feature scaling, class-balancing via SMOTE^23^, feature selection, and SVM^24^ classification into a unified framework. This ensures that SMOTE and feature selection are applied strictly within each cross-validation fold, preventing any information from the test set from influencing model training. In contrast, our previous study17 applied these steps sequentially outside of the cross-validation loop. Furthermore, the current study employs nested cross-validation (five-fold outer loop for evaluation, three-fold inner loop for hyperparameter tuning via grid search), whereas the previous study used single-layer five-fold cross-validation without hyperparameter optimization. The feature extraction step and patient-level aggregation (mean pooling) remain the same between the two studies. Moreover, while the previous study focused on differentiating between responders and nonresponders to NAC, the present work shifts the endpoint to a clinically meaningful outcome: predicting three-year overall survival in LABC patients based solely on pre-treatment QUS imaging. This shift not only enhances the translational relevance of the model but also aligns the prediction task more closely with long-term patient outcomes.

## Results

### Patient, tumor, and treatment characteristics

Table 1 summarizes the demographic and clinical characteristics of patients with locally advanced breast cancer receiving neoadjuvant chemotherapy. The mean age of the research participants was 51 ± 11 years. The average tumor size among patients ranged from 5.18 ± 2.79 cm at the beginning of treatment to 2.71 ± 3.48 cm afterward. Of the 172 patients, 34 were categorized as non-survivors, while 138 were classified as survivors three years after treatment initiation. Histological analysis indicates that invasive ductal carcinoma (IDC) accounted for 91% of the patients, invasive lobular carcinoma (ILC) for 3%, and invasive mixed carcinoma (IMC) for the remaining 6%. 9% of patients had unreported tumors, while 6% had Grade I tumors, 38% had Grade II tumors, and 47% had Grade III tumors. Following systemic therapy, 42% of patients received ACT (a combination of Adriamycin, Cyclophosphamide, and Paclitaxel [Taxol]), 21% received FECD (5-Fluorouracil, Epirubicin, Cyclophosphamide, and Docetaxel), and 37% received other chemotherapy regimens. Molecular subtypes such as ERBB2+ (ER-, PR-, HER2+), triple-negative (ER-, PR-, HER2-), Luminal-A (ER + and/or PR+, HER2-), Luminal-B (ER + and/or PR+, HER2+), were used to distinguish between tumors. The molecular subtype distribution within the survivors was as follows: 12% were categorized as ERBB2+, 25% triple negative, 41% as Luminal-A, 22% as Luminal-B. The molecular subtypes of the non-survivors were as follows: 12% were categorized as ERBB2+, 29% as triple negative, 32% as Luminal-A and 26% as Luminal-B.

**Table 1:** Demographic and clinical characteristics of patients with locally advanced breast cancer receiving neoadjuvant chemotherapy.

	NS (*N* = 34)	S (*N* = 138)	All (*N* = 172)
Age (y)	52 ± 11	51 ± 11	51 ± 11
**Reproductive Stage**			
Postmenopausal (%)	38	49	47
Premenopausal (%)	59	37	41
Perimenopausal (%)	3	12	10
Initial Tumor Size (cm)	6.77 ± 2.98	4.79 ± 2.61	5.18 ± 2.79
**Histology**			
IDC (%)	91	91	91
ILC (%)	3	4	3
IMC (%)	6	6	6
**Tumor Grade**			
Grade I (%)	3	7	6
Grade II (%)	53	34	38
Grade III (%)	44	48	47
Not Reported (%)	0	11	9
**Molecular Subtype**			
ERBB2+ (%)	12	12	12
Triple Negative (%)	29	25	26
Luminal-A (%)	32	41	39
Luminal-B (%)	26	22	23
**Treatment**			
ACT (%)	44	41	42
FECD (%)	15	22	21
Other (%)	41	36	37
Residual Tumor Size (cm)	4.09 ± 5.24	2.37 ± 2.82	2.71 ± 3.48

IDC = invasive ductal carcinoma; ILC = invasive lobular carcinoma; IMC = invasive mixed carcinoma; ACT = adriamycin, cyclophosphamide, paclitaxel (taxol); FECD = 5-fluorouracil, epirubicin, cyclophosphamide, docetaxel.

**Table 2:** Model performance on the hold-out (unseen) patient dataset.

QUS	Patient	Precision (%)	Recall (%)	F1-score (%)	Balanced Accuracy (%)	Accuracy (%)	AUC
AAC	Non-survivor	75 [47, 87]	60 [28, 90]	67 [39, 85]	78 [61, 93]	88 [81, 94]	0.94 [0.86, 1.00]
	Survivor	91 [83, 98]	95 [89, 97]	93 [88, 97]			
ASD	Non-survivor	67 [36, 98]	60 [24, 92]	63 [31, 88]	76 [57, 92]	86 [74, 97]	0.74 [0.56, 0.90]
	Survivor	91 [79, 100]	93 [81, 100]	92 [83, 100]			
MBF	Non-survivor	57 [22, 89]	80 [45, 100]	67 [33, 90]	83 [65, 98]	86 [74, 95]	0.77 [0.51, 0.94]
	Survivor	95 [85, 100]	86 [76, 95]	90 [82, 96]			
SI	Non-survivor	38 [5, 68]	50 [18, 82]	43 [10, 65]	65 [48, 81]	76 [64, 85]	0.74 [0.56, 0.90]
	Survivor	87 [76, 95]	81 [69, 90]	84 [75, 90]			
SS	Non-survivor	55 [18, 93]	60 [28, 93]	57 [23, 81]	74 [58, 91]	83 [71, 92]	0.84 [0.66, 0.96]
	Survivor	90 [79, 99]	88 [79, 95]	89 [81, 95]			
COMBINED	Non-survivor	60 [25, 90]	60 [25, 90]	60 [25, 82]	75 [57, 91]	85 [73, 94]	0.81 [0.63, 0.94]
	Survivor	90 [80, 98]	90 [79, 98]	90 [82, 97]			

Values reported as point estimate [95% confidence interval]. Confidence intervals were computed via 1000 bootstrap resamples (with replacement) of the hold-out test set (*n* = 52; 10 non-survivors, 42 survivors). Balanced accuracy, accuracy, and area under the curve (AUC) are overall model metrics shown only on the non-survivor row. AAC denotes the best-performing model. COMBINED denotes the multi-parametric model with feature-level fusion of all five QUS maps.

### QUS parametric maps

**Fig. 1:**
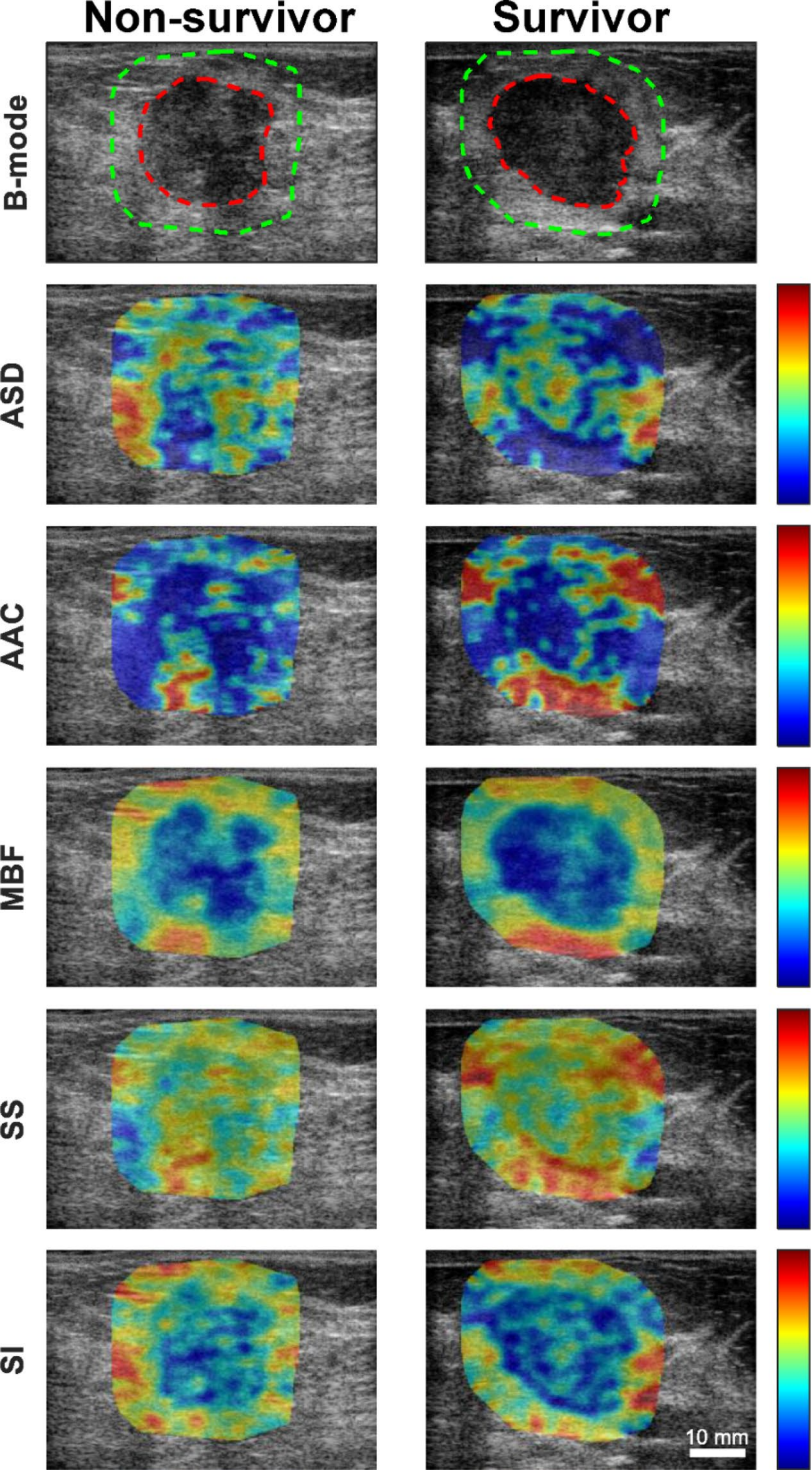
Representative B-mode and QUS parametric images (ASD, AAC, MBF, SS, and SI) showing tumor core (red contour) and margin regions (green contour) in a non-survivor and a survivor at pre-treatment. Each map uses blue (minimum) to red (maximum) scale. The parametric value ranges are as follows: ASD: 1–176 μm, AAC: 17–173 dBr/cm³, MBF: − 11–29 dBr, SS: − 6–2 dBr/MHz, and SI: − 5–40 dBr. The unit dBr (decibels relative) expresses the ratio of measured acoustic values relative to a calibration reference phantom level.

Figure 1 shows QUS parametric maps of ASD, AAC, MBF, SS, and SI, which are overlaid on B-mode ultrasound images from representative non-surviving and surviving patients prior to treatment. The B-mode image shows a hypo-intense tumor mass which is surrounded by fibroglandular tissue that appears more hyper-intense. Parametric maps from quantitative ultrasound reveal new information about microstructural tumor characteristics through unique textural patterns^25,26^.

### Classification performance

**Fig. 2:**
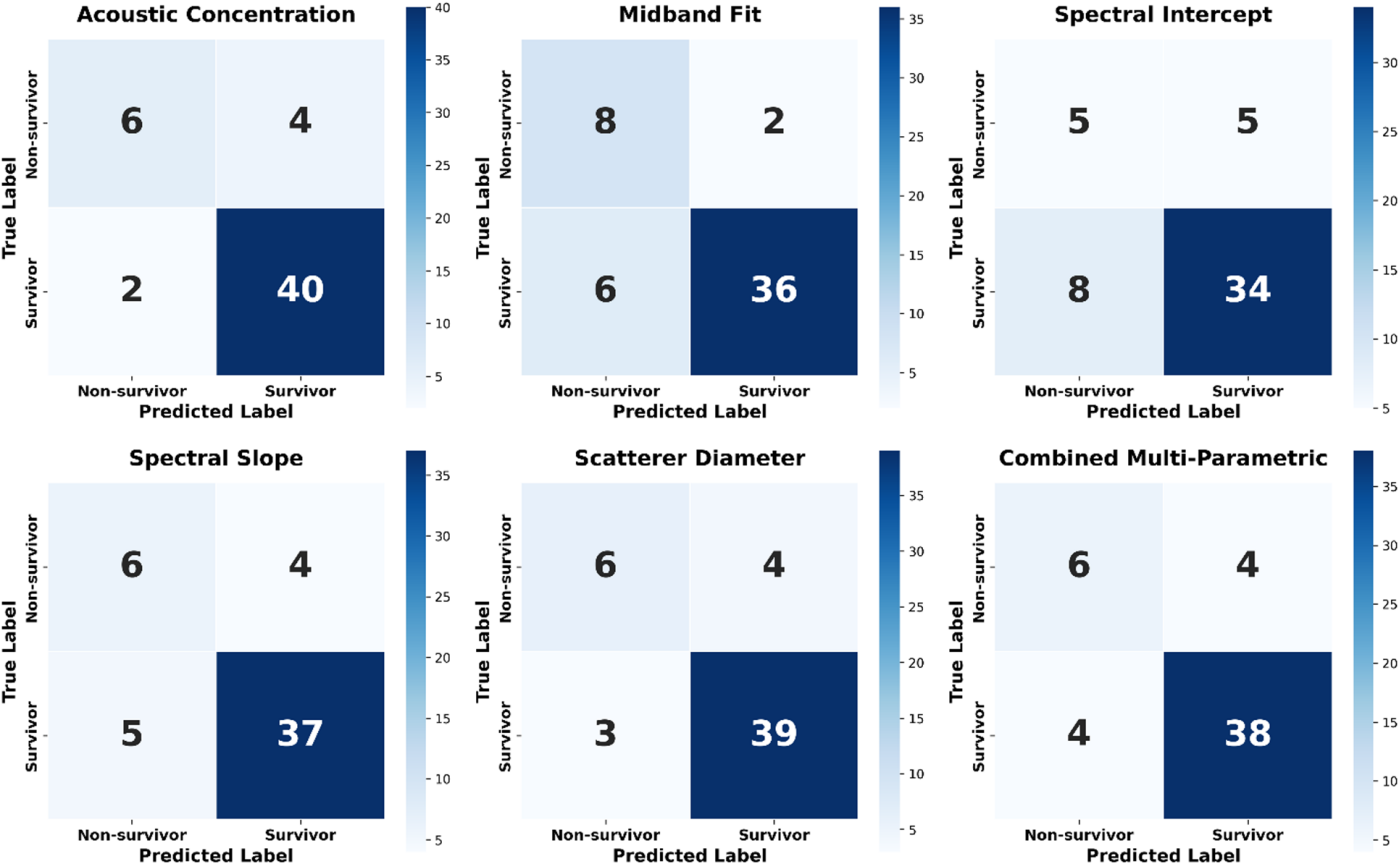
Confusion matrices of three-year survival prediction on the unseen dataset.

Figure 2 shows the confusion matrices of three-year survival on the unseen dataset. For the acoustic concentration, the proposed pipeline is capable of precisely predicting 40 out of 42 survivors. Out of ten non-survivors, six are accurately predicted.

**Fig. 3:**
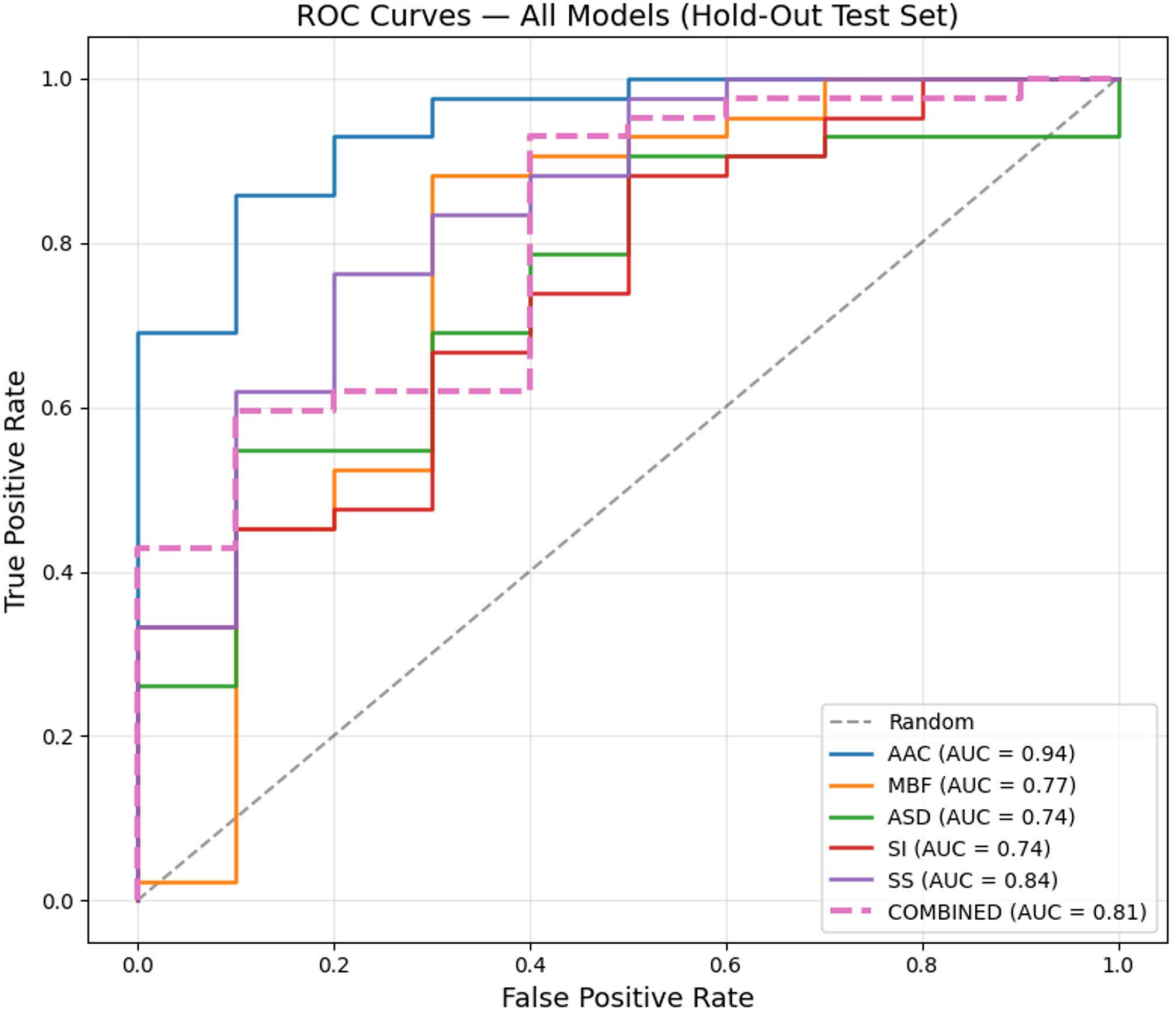
Receiver operating characteristic (ROC) curves for all five QUS parametric maps (AAC, ASD, MBF, SI, SS) on the hold-out test set (*n* = 52). The AUC for each model is shown in the legend. The diagonal dashed line indicates the performance of a random classifier (AUC = 0.5). AAC achieved the highest AUC of 0.94, demonstrating excellent discriminative ability between survivors and non-survivors at three years.

Table 2 shows the results of three-year survival on the unseen patients’ dataset. The dataset was split using a stratified 70/30 train-test split, yielding 120 patients for training and 52 patients for the hold-out test set. Among all QUS parametric images, the model developed based on Average Acoustic Concentration (AAC) parametric images performed the best in survival prediction before treatment initiation, with precision, recall, and F1-Scores of 75%, 60%, and 67% for non-surviving patients and 91%, 95%, and 93% for surviving patients. The pipeline achieved a balanced accuracy of 78% on the unseen patients’ dataset. All performance metrics in Table 2 are reported as point estimates followed by their 95% confidence intervals (CIs), computed via 1,000 bootstrap resamples (with replacement) from the hold-out test set. The wider CIs observed for the non-survivor class reflect the limited size of this minority class (*n* = 10) within the test cohort. Receiver operating characteristic (ROC) analysis was performed on the hold-out test set for all five quantitative ultrasound (QUS) parametric maps and the combined multi-parametric model. Figure 3 presents the ROC curves and their corresponding area under the curve (AUC) values, providing a threshold-independent measure of the models’ discriminative capabilities. The AAC model achieved the highest AUC of 0.94 (95% CI: 0.86–1.00), demonstrating superior predictive performance relative to the other QUS parameters. The SS model achieved the second-highest AUC of 0.84 (95% CI: 0.66–0.96), followed by the combined multi-parametric model at 0.81 (95% CI: 0.63–0.94), MBF at 0.77 (95% CI: 0.51–0.94), ASD at 0.74 (95% CI: 0.56–0.90), and SI at 0.74 (95% CI: 0.56–0.90). Notably, the combined model did not outperform the isolated AAC model. This underperformance likely stems from overfitting induced by the high-dimensional concatenated feature space (approximately 180,000 features) relative to the limited training cohort (*n* = 120 patients). For comprehensive comparison, the AUC values for all parametric maps and the combined model are summarized alongside the other performance metrics in Table 2.

## Discussion and conclusion

This study introduced an integrated deep-learning pipeline for predicting LABC three-year survival after neoadjuvant chemotherapy using QUS imaging at pre-treatment. It has been hypothesized that biological alterations in tumor microstructure and spatial heterogeneity are associated with QUS-detected responses in tumors undergoing treatment. To identify tumor response to treatment early and during the course of treatment, several preclinical and clinical QUS studies were conducted^21,26–28^. In these studies, QUS spectral parameters such as MBF, SS, and SI showed a strong correlation with tumor response to treatment. Scatterer properties, such as scatterer size and scatterer acoustic concentration, are linked to these spectral parameters^16,29^. The ASD and AAC have also been used to track tumor response in LABC patients undergoing NAC. These metrics are calculated from the ultrasound backscatter coefficient by fitting a spherical Gaussian model to the measured backscatter coefficient^29^. Additionally, it has been demonstrated that textural characteristics of QUS parametric maps, including contrast, correlation, energy, and homogeneity, can effectively predict breast cancer patients’ responses to therapy.^26,30^

The developed pipeline was evaluated using four performance metrics on the unseen dataset. These include precision, recall, F1-score, and balanced accuracy. The percentage of correctly classified positive cases out of all cases predicted to be positive is known as precision or positive predictive value (PPV). It measures the classifier’s ability to avoid making incorrect positive predictions. Recall, which is the percentage of correctly classified positive cases out of all actual positive cases, is also referred to as sensitivity or true positive rate. It measures the classifier’s ability to identify positive cases. F1-score, which is the harmonic mean of precision and recall, offers a balanced measurement when there is an imbalance between positive and negative cases. To account for the imbalance in class sizes, balanced accuracy, which measures the mean of the per-class accuracies, was calculated. It provides a comprehensive evaluation of the classifier’s performance across different classes. The results of the pipeline on an unseen dataset demonstrated that the average acoustic concentration performed the best in survival prediction, with precision, recall, and F1-score values of 75%, 60%, and 67% for the non-surviving patients and 91%, 95%, and 93% for the surviving patients, respectively. AAC, which refers to the average density of scatterers in a given volume of tissue, is derived from the backscattered radiofrequency (RF) signals during ultrasound imaging. Previous studies have shown that the AAC can differentiate between benign and malignant tumors and quantify cell death in breast cancer models^25,31^. The proposed pipeline achieved a balanced accuracy of 78%, with an overall accuracy of 88%, indicating reliable performance across both survivor and non-survivor groups despite class imbalance.

A recall rate of 95% for the survivor class suggests that this pipeline can effectively identify survivors, thereby minimizing the risk of false negatives, which is crucial in clinical settings. The pipeline’s high precision of 91% for the survivor class indicates that it produces few false positives among predicted survivors, meaning most patients identified as survivors are indeed true survivors. These combined metrics demonstrate the strength of the developed pipeline by capturing nearly all true positive cases while maintaining minimal misclassification rates. The high F1 score indicates that the pipeline maintains strong performance, striking a balance between sensitivity and precision.

Predicting three-year survival is biologically linked to, yet clinically distinct from, predicting initial treatment response. Although patients who respond favorably to neoadjuvant chemotherapy frequently achieve long term survival, particularly those reaching a pathological complete response (pCR), overall survival remains a multifactorial endpoint. It is heavily influenced by variables beyond the initial tumor response, including subsequent disease recurrence, distant metastasis, patient comorbidities, and the efficacy of subsequent therapeutic interventions. Consequently, forecasting overall survival is an inherently more complex predictive task. This increased clinical complexity explains the performance difference observed between our two studies. Specifically, our prior response prediction model^21^ achieved an 88% balanced accuracy, whereas the current survival prediction model reached a 78% balanced accuracy. Remarkably, average acoustic concentration (AAC) emerged as the most robust predictor across both independent tasks. This consistency indicates that the microstructural tissue properties quantified by AAC encode fundamental information regarding baseline tumor aggressiveness, which ultimately governs both immediate treatment sensitivity and long-term patient outcomes.

The success of the model largely comes down to the specific quantitative features it evaluates. As we demonstrated previously^21^, average acoustic concentration (AAC) serves as the strongest predictor of three year survival and effectively distinguishes between patients who respond to treatment and those who do not. These findings indicate that the physical properties of tissue microstructure captured by AAC, particularly scatterer density and spatial distribution, offer valuable predictive and prognostic insight. We believe AAC performs so well because it is highly sensitive to microscopic structural features, such as cellular density, nuclear size, and overall tissue architecture at the scale of tens of microns. Unlike B mode echogenicity, which relies on system dependent signal amplitudes, AAC is calculated by fitting a spherical Gaussian model to the radiofrequency (RF) backscatter coefficient. Consequently, AAC reflects the true physical properties of the tissue rather than the settings of the imaging system. Because tumors with different microstructural compositions (like varying degrees of necrosis or cellularity) yield distinct AAC values, a patient’s likelihood of a positive outcome is closely tied to the baseline aggressiveness of the tumor. The well-established link between achieving a pathological complete response (pCR) and improved long-term survival further reinforces this intrinsic relationship.

It is important to note that the pipeline was less accurate at classifying patients who did not survive. There are a few key reasons for this. First, our dataset was highly imbalanced, with 138 survivors compared to just 34 patients who passed away, which is roughly a four to one ratio. Although we used the Synthetic Minority Over-sampling Technique (SMOTE) to help balance the training data, the small number of actual mortality cases makes it difficult for the model to learn every possible pattern. Additionally, the holdout test set included only ten patients from this group, naturally making the performance estimates for these cases a bit more variable. Second, mortality can stem from a wide variety of causes, such as disease progression, distant metastasis, treatment complications, or other health issues. These different clinical pathways likely create a very diverse set of quantitative ultrasound (QUS) signatures, especially when compared to the much more consistent profiles seen in surviving patients. Even with this lower accuracy for identifying mortality cases, the 95% recall rate for survivors proves that the pipeline is highly effective at identifying patients who are likely to do well. This reliability offers real clinical value for treatment planning. Moving forward, we plan to improve our detection of high-risk patients by studying much larger cohorts and integrating different types of diagnostic data.

Compared to our previously published model^21^, which employed a single-layer 5-fold cross-validation and an SVM classifier, the current study introduces a more statistically robust pipeline. Although both approaches share key components, including image preprocessing, deep feature extraction, patient-level aggregation, and SMOTE for class balancing, our previous method relied on K-fold splitting and omitted hyperparameter optimization. While computationally efficient, it has limitations in terms of consistently generalizing across patient cohorts. In contrast, this study employs a nested cross-validation approach, in which an outer loop provides an objective assessment of model performance while an inner loop optimizes hyperparameters using the grid search technique. This design adheres to best practices for reproducible machine learning in biomedical applications by minimizing information leakage and lowering the chance of overfitting^32–34^. As such, our developed pipeline represents a significant improvement over our earlier model, providing improved clinical reliability and generalizability for patient classification.

Understanding the three-year survival rates of LABC patients is crucial for several reasons, including prognosis estimation, treatment planning, and informed decision-making. Clinicians use this three-year benchmark to evaluate the efficacy of treatment interventions and disease progression. Moreover, it helps clinicians estimate likely outcomes based on historical patterns, enabling risk stratification and personalized treatment planning. First, knowing the three-year overall survival rates may help in creating a framework for predicting the prognosis of LABC patients. Studies have shown that neoadjuvant chemotherapy (NAC) can significantly impact therapy outcomes by facilitating earlier surgical intervention and improving rates of breast-conserving surgery^35,36^. A significant number of patients who have favorable outcomes after NAC, represented by a pathological complete response (pCR), experience improved overall survival rates^37^. Additionally, survival data derived from subgroups (e.g. hormone receptor status and subtype classification) can refine expectations and recommendations, especially since changes in biological markers have been shown to significantly affect survival outcomes^30,38^. Survival rates are also a crucial factor in treatment planning, as they guide the selection of treatment regimens. For instance, a phase II trial examining primary systemic therapy for LABC patients demonstrated the prognostic value of early clinical response, highlighting the need for personalized treatment approaches^39^. When oncologists are aware of survival statistics, they can decide whether to be more aggressive in their approach or provide modified treatment plans when they are not satisfied with the initial response to treatment. Furthermore, understanding the three-year survival scenario improves communication between physicians and their patients. When patients are informed about their prognosis, they can effectively engage in shared decision-making processes, considering their preferences for treatment in conjunction with their expectations of the outcomes. This is particularly pertinent in LABC, where decisions may be influenced by psychological and emotional consequences of treatment choices, including surgical options such as mastectomy versus breast-conserving surgery^40^.

In conclusion, this study presented a deep learning pipeline that combined quantitative ultrasound (QUS) imaging and machine learning to predict three-year survival rates for patients with locally advanced breast cancer (LABC) who received neoadjuvant chemotherapy. The pipeline was trained on five quantitative ultrasound maps at the pre-treatment stage. The average acoustic concentration was the most predictive feature, achieving a recall and precision of 95% and 91%, respectively, for the survivor class. This work demonstrates that QUS shows potential as a non-invasive biomarker for differentiating between LABC survivors and non-survivors at the pre-treatment stage, pending further validation on larger, multi-institutional cohorts. Future work will involve the multimodal integration of QUS with clinical and genomic data, as well as prospective validation across larger, multi-institutional cohorts to assess the robustness of this approach in clinical settings.

## Methods

### Study protocol

The study protocol is described in detail in our previous work^21^. Briefly, the study was conducted in accordance with the rules and regulations established by the Sunnybrook Health Sciences Centre’s institutional research ethics board and was registered with ClinicalTrials.gov (NCT00437879). All experimental protocols were reviewed and approved by the Sunnybrook Research Institute research ethics board before commencing the study. All patients were enrolled with informed consent. The trial enrolled 172 women who were diagnosed with LABC and scheduled to undergo NAC followed by surgery. As part of their standard of care, all patients had a core needle biopsy before treatment to confirm a cancer diagnosis, histological subtype, and hormone receptor status to identify the tumor molecular subtype. As part of the institutional standard of care for such patients, magnetic resonance images were collected using a 1.0-T clinical MRI (GE Healthcare, Waukesha, WI) to determine the initial tumor size prior to treatment. Ultrasound scans were performed shortly before patients started therapy. Three-year survival was defined using overall survival (OS), the gold standard endpoint. OS was measured from the time of the initiation of neoadjuvant chemotherapy until death from any cause.

### QUS parametric maps generation

The RF-enabled Sonix RP system (Analogic Medical Corp., Vancouver, Canada) equipped with an L14-5/60 transducer (center frequency of 6 MHz with a − 6 dB bandwidth of 3–8 MHz) was used to collect the ultrasound RF data before the treatment. Four to seven image planes, approximately one centimetre apart, were collected for each patient. A region of interest (ROI) corresponding to the tumour was manually contoured under the supervision of a specialist oncologist. The standardization procedures and the computation of quantitative ultrasound parameters using custom MATLAB-based software (MathWorks, Natick, MA, version 2025b) are detailed in previous work^14^. For every ultrasound scan line, the Fourier transform of the Hanning-gated RF signal was calculated and averaged for the examined regions to determine the mean power spectrum. The latter was normalized using a reference phantom technique to remove the effects of the transducer diffraction pattern, attenuation^41^, and the system transfer function. Glass beads ranging in diameter from 5 to 30 µ*m* were embedded in a uniform background of microscopic oil droplets in gelatin to create the reference phantom (Medical Physics Department, University of Wisconsin, USA). Linear regression analysis was used to estimate the MBF, SS, and SI parameters within the transducer’s -6 dB bandwidth^15,16^. ASD and AAC parameters were obtained by fitting a spherical Gaussian form factor model to the estimated backscatter coefficient^17,42^. Finally, a sliding window technique was employed to produce colour-coded parametric maps for every QUS parameter. Each region of interest (ROI), which included the tumor core and 5-mm margin, was segmented into square analysis blocks spanning 10 ultrasonic wavelengths, with neighboring blocks overlapping by 94% in both axial and lateral dimensions^43,44^.

### Machine learning pipeline

**Fig. 4:**
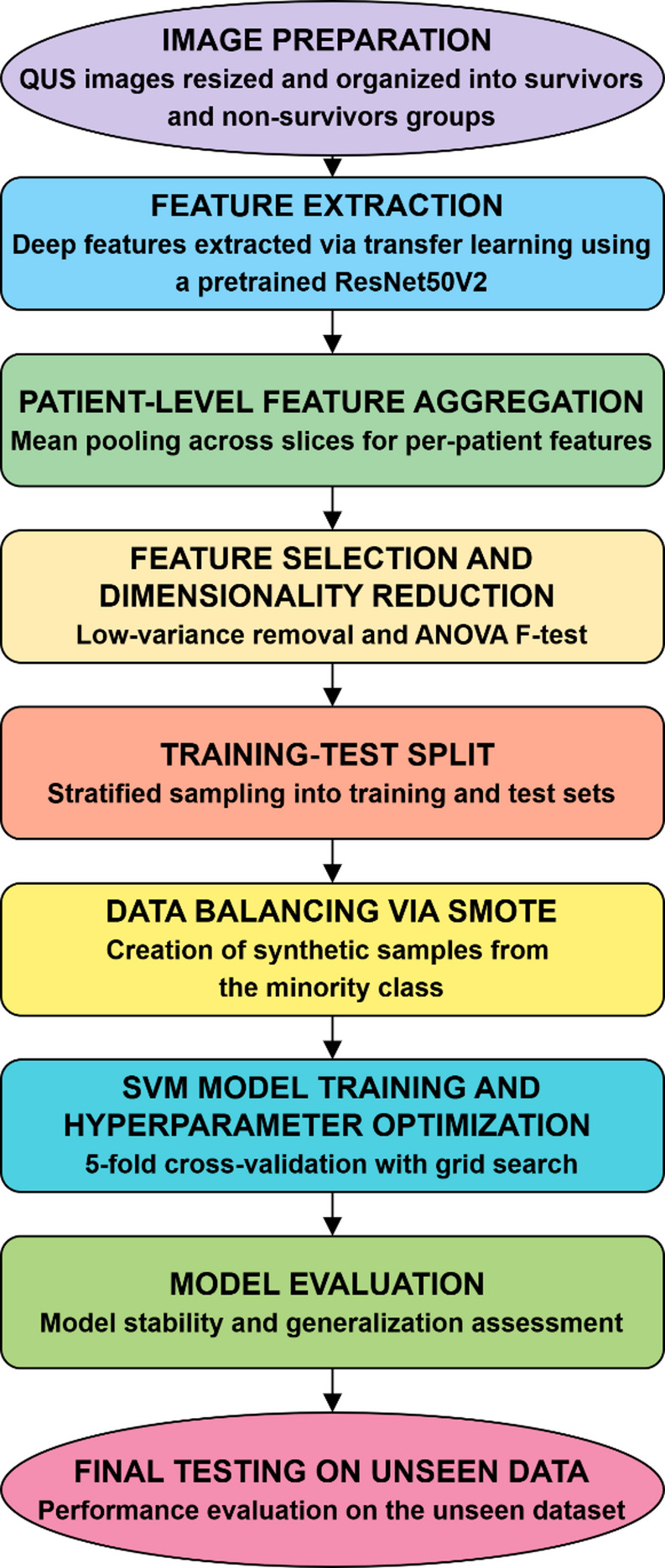
Overview of the machine learning pipeline for the Prediction of Three-Year Survival in Locally Advanced Breast Cancer Patients Using Quantitative Ultrasound Imaging. The pipeline includes resizing images and organizing them into two groups (survivors and non-survivors). This is followed by extracting deep features using a pre-trained ResNet50V2 model. The extracted features undergo patient-level aggregation followed by dimensionality reduction before being split into training and testing sets. The SMOTE technique handles class imbalance, while the SVM classifier is trained via 5-fold cross-validation with hyperparameter tuning. The model’s performance is assessed through cross-validation and an independent test set using performance metrics.

An end-to-end machine learning pipeline (Fig. 4) was created to classify three-year survival outcomes based on pre-treatment quantitative ultrasound images of patients with locally advanced breast cancer. The process started with image preparation, in which the QUS images were classified into two diagnostic classes (survivor or non-survivor), cropped and resized to 224 × 224 pixels to remove the excess background. Deep feature extraction was applied to all QUS images using a pretrained ResNet50V2^22,45^ a convolutional neural network (CNN) on the ImageNet database^46^. The ResNet50V2 model was used strictly as a fixed feature extractor with all weights frozen at their pre-trained ImageNet values; no fine-tuning or weight updates were performed. The extracted features were then aggregated into a single representative feature vector per patient using mean pooling. This was followed by feature selection, where low variance (zero) features were removed, and the best 13 features ($\:13=\sqrt{172}\:,\:172\:$ being the number of patients) were selected using SelectKBest algorithm^47^. SelectKBest performs univariate statistical tests using the ANOVA F-test, which ranks all features by importance. Features with the highest score are kept. After the features were extracted and selected, the dataset was split into training and testing sets, stratified to maintain class balance across the classes. Within the training data, the class imbalance was addressed by using the Synthetic Minority Over-sampling Technique (SMOTE)^23^ to create synthetic minority instances. SMOTE addresses class imbalance by generating synthetic examples for the minority group. For each sample within this smaller class, the algorithm identifies its k nearest neighbors (typically five by default) in the feature space. It then creates new synthetic data points by interpolating along the segments that connect the original sample to these neighbors. This approach produces realistic new instances that enrich the training set without merely duplicating existing data, thereby preventing the classifier from developing a bias toward the majority class. All preprocessing and classification steps, including feature scaling (StandardScaler), SMOTE oversampling, feature selection (SelectKBest), and SVM classification, are integrated into a single unified pipeline. This ensures that SMOTE and feature selection are applied strictly within each cross-validation fold, preventing any information from the test set from influencing model training. A Support Vector Machine (SVM) classifier^24^ was then used to train the model using hyperparameter tuning within a nested cross validation strategy. This strategy utilized a five-fold outer loop for performance estimation and a three-fold inner loop for hyperparameter optimization via grid search. The SVM classifier was configured with probability set to true, enabling Platt scaling to produce probability estimates. Classification used the default decision threshold of 0.5. Specifically, a patient is classified as a survivor if the predicted probability for the survivor class exceeds 0.5, and as a mortality case otherwise. The two-stage approach of deep feature extraction followed classification was chosen for several reasons. First, with 172 patients and a class imbalance (138 survivors vs. 34 non-survivors), end-to-end fine-tuning of a deep CNN would be prone to overfitting. By using a pre-trained ResNet50V2 as a fixed feature extractor (with ImageNet weights frozen), we leverage the rich feature representations learned from millions of natural images without requiring a large medical imaging dataset for training. Second, each patient has 4–7 image planes; by extracting features from each image and then aggregating them via mean pooling at the patient level, we ensure the classifier operates on patient-level feature vectors, preventing data leakage that could occur if individual images from the same patient appeared in both training and testing sets. Third, using an SVM classifier on extracted features allows for systematic hyperparameter optimization via grid search. This two-stage transfer learning approach is well-established in the medical imaging literature for small-to-moderate dataset sizes.

Model performance was assessed across five folds for each evaluation metric, including balanced accuracy, sensitivity, specificity, precision, and F1-score. The best model was retrained on the training dataset and evaluated on the unseen (holdout) test dataset using the evaluation metrics above and visualized using the confusion matrices and ROC curves. The AUC was calculated from the support vector machine (SVM) probability outputs, calibrated via Platt scaling, yielding a threshold-independent measure of discriminative ability. To quantify the uncertainty of the performance metrics on the hold-out test set (*n* = 52), 95% confidence intervals were estimated using bootstrap resampling. Specifically, the test cohort was resampled with replacement 1,000 times, and each evaluation metric was recalculated for every bootstrap iteration. The 2.5th and 97.5th percentiles of the resulting empirical distributions were extracted to define the lower and upper bounds of the 95% CIs. This pipeline guarantees accurate model calibration, fair evaluation, and reliable results for QUS imaging-based imbalanced survival classification.

## Data Availability

The data that support the findings of this study are available from Sunnybrook Research Institute, but restrictions apply to the availability of these data, which were used under license for the current study, and so are not publicly available. Data are, however, available from the authors upon reasonable request and with permission of Dr. Gregory J. Czarnota, Sunnybrook Research Institute.
